# Border ownership-dependent tilt aftereffect for shape defined by binocular disparity and motion parallax

**DOI:** 10.1152/jn.00111.2019

**Published:** 2019-03-27

**Authors:** Reuben Rideaux, William J. Harrison

**Affiliations:** ^1^Department of Psychology, University of Cambridge, Cambridge, United Kingdom; ^2^Queensland Brain Institute, The University of Queensland, Brisbane, Queensland, Australia

**Keywords:** binocular disparity, border ownership cells, cue integration, figure-ground segmentation, motion parallax

## Abstract

Discerning objects from their surrounds (i.e., figure-ground segmentation) in a way that guides adaptive behaviors is a fundamental task of the brain. Neurophysiological work has revealed a class of cells in the macaque visual cortex that may be ideally suited to support this neural computation: border ownership cells (Zhou H, Friedman HS, von der Heydt R. *J Neurosci* 20: 6594–6611, 2000). These orientation-tuned cells appear to respond conditionally to the borders of objects. A behavioral correlate supporting the existence of these cells in humans was demonstrated with two-dimensional luminance-defined objects (von der Heydt R, Macuda T, Qiu FT. *J Opt Soc Am A Opt Image Sci Vis* 22: 2222–2229, 2005). However, objects in our natural visual environments are often signaled by complex cues, such as motion and binocular disparity. Thus for border ownership systems to effectively support figure-ground segmentation and object depth ordering, they must have access to information from multiple depth cues with strict depth order selectivity. Here we measured in humans (of both sexes) border ownership-dependent tilt aftereffects after adaptation to figures defined by either motion parallax or binocular disparity. We find that both depth cues produce a tilt aftereffect that is selective for figure-ground depth order. Furthermore, we find that the effects of adaptation are transferable between cues, suggesting that these systems may combine depth cues to reduce uncertainty (Bülthoff HH, Mallot HA. *J Opt Soc Am A* 5: 1749–1758, 1988). These results suggest that border ownership mechanisms have strict depth order selectivity and access to multiple depth cues that are jointly encoded, providing compelling psychophysical support for their role in figure-ground segmentation in natural visual environments.

**NEW & NOTEWORTHY** Figure-ground segmentation is a critical function that may be supported by “border ownership” neural systems that conditionally respond to object borders. We measured border ownership-dependent tilt aftereffects to figures defined by motion parallax or binocular disparity and found aftereffects for both cues. These effects were transferable between cues but selective for figure-ground depth order, suggesting that the neural systems supporting figure-ground segmentation have strict depth order selectivity and access to multiple depth cues that are jointly encoded.

## INTRODUCTION

Our natural visual environments are complex and often cluttered with objects. To interact with our surrounds appropriately, therefore, objects must be segmented from other objects and their backgrounds, and their position in depth must be inferred from often fragmented and ambiguous cues such as binocular disparity, motion parallax, and texture. Achieving such so-called “figure-ground segmentation” with the speed and automaticity necessary to effectively function within the environment is nontrivial; understanding how the brain accomplishes this remains of fundamental importance in neuroscience.

**Fig. 1.**
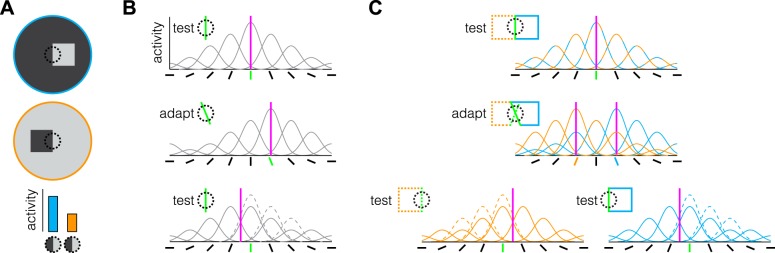
Illustrations of border ownership neurons and putative explanations of tilt aftereffects. *A*: the same dark-light edge within the receptive field of certain orientation-tuned neurons (dotted circle) will evoke different levels of activity depending on whether the light region is part of a “figure” and the dark region part of the “ground” (cyan) or vice versa (orange). *B*: the perceived orientation (magenta line) of a vertical bar (green) is based on the population response of multiple orientation-tuned cells (*top*). Prolonged exposure to a bar tilted clockwise from vertical reduces the responsiveness of cells tuned to this orientation (i.e., adaptation; *middle*). After adaptation, viewing a vertical bar will now produce a population response that is biased away from the adapted orientation, i.e., a tilt aftereffect (*bottom*), because of the attenuated response of the cells (dashed lines). *C*: by extending the vertical bar such that it becomes the border of a figure, on the left (dotted orange) or on the right (cyan), the bar will now evoke activity from separate populations of border ownership cells (*top*). Prolonged alternating viewing of borders tilted clockwise and anticlockwise from vertical that belong to the left and right figures, respectively, will produce border ownership-dependent adaptation in separate populations of cells with receptive fields in the same retinotopic location (*middle*). Now, when a vertical border is viewed it will produce a population response that is biased away from the adapted tilt orientation of the figure it belongs (*bottom*).

Neurophysiological work has revealed a class of cells in the macaque primary visual cortex that has been implicated in figure-ground segmentation. These cells, known as border ownership cells (Zhou et al. 2000), are tuned to oriented edges, like many neurons in the primary visual cortex (Hubel and Wiesel 1959). However, unlike most orientation-tuned cells, the activity of border ownership cells is contingent on whether or not the edge belongs to the border of an object (Zhou et al. 2000). For instance, the same light-dark edge presented within the receptive field of a neuron could produce a larger increase in firing rate if the light region was part of a distinct “figure” positioned on a dark “background” than vice versa (Fig. 1*A*). Critically, this contingency is active even when the object extends far beyond the classic receptive field of the cell, suggesting that border ownership cells are connected through a network that can identify borders that are common to an object. This distinctive characteristic is ideally suited for binding the borders of an object in order to segment it from the background and facilitate other figure-ground mechanisms to retrieve those borders as a whole.

A behavioral correlate of the contingency that characterizes border ownership cells can be demonstrated psychophysically in humans with a modification of the classic tilt aftereffect paradigm (Fig. 1*B*). This was elegantly demonstrated by von der Heydt et al. (2005), who presented two luminance-defined adaptor figures alternating in time. One object was presented to the left of center, and the other object was presented to the right of center. Importantly, the inner borders of these objects intersected in space but were of opposite tilt. When a solitary test line was subsequently presented at the intersection, no tilt aftereffect was observed, presumably because the effects of the adapting edges were balanced. In contrast, if the test line was extended to form a figure to the left or the right of fixation, a tilt aftereffect was observed that was repulsed away from the border of the adaptor on the side to which the test line extended (Fig. 1*C*).

Objects in our natural environment are seldom defined by a simple luminance discontinuity; their borders are often signaled by multiple depth cues such as motion parallax and binocular disparity. Thus for the neural systems of border ownership to effectively support figure-ground segmentation, they must have access to information from multiple depth cues. Furthermore, to establish the depth order of borders these systems must have depth order selectivity. Finally, depth cues are noisy and often unreliable; to yield accurate inferences, psychophysical evidence suggests that for some tasks the brain combines information from multiple depth cues (Ernst and Bülthoff 2004). Thus if depth cues are used by the neural systems supporting border ownership, determining whether these cues are jointly encoded will reveal if similar mechanisms for uncertainty reduction may be utilized in figure-ground segmentation.

To test whether the neural systems supporting border ownership use depth cues, and whether they are selectivity tuned to a particular depth order, we measured border ownership-dependent tilt aftereffects following adaptation to figures defined by two primary depth cues: *1*) motion parallax and *2*) binocular disparity. We found that both depth cues produce a tilt aftereffect that is selective for depth order. Moreover, we found that the effect of adaptation is transferable between cues. These results indicate that the neural systems supporting perceptual figure-ground segmentation have strict depth order selectivity and access to multiple depth cues that are jointly encoded.

## METHODS

### 

#### Participants.

Observers were recruited from the University of Cambridge, had normal or corrected-to-normal vision, and were screened for stereo deficits with a fine discrimination task (just-noticeable difference < 0.5 arcmin). Eight right-handed human adults participated in each of the two experiments (*experiment 1*: 4 men, 4 women, 26.3 ± 5.6 yr; *experiment 2*: 3 men, 5 women, 26.7 ± 4.9 yr). Five participants participated in both experiments, including one author (R. Rideaux). With the exception of R. Rideaux, all participants were naive to the aims of the experiment. Experiments were reviewed and approved by the University of Cambridge ethics committee; all observers provided written informed consent.

#### Apparatus and stimuli.

Stimuli were generated in MATLAB (The MathWorks, Natick, MA) with Psychophysics Toolbox and Eyelink Toolbox extensions (Brainard 1997; Cornelissen et al. 2002; Pelli 1997; see http://psychtoolbox.org/). Binocular presentation was achieved with a pair of Samsung 2233RZ LCD monitors (120 Hz, 1,680 × 1,050) viewed through front-silvered mirrors in a Wheatstone stereoscope configuration. The viewing distance was 50 cm, and the participants’ head positions were stabilized with an eye mask, a headrest, and a chin rest. Eye movement was recorded binocularly at 1 kHz with an EyeLink 1000 (SR Research).

**Fig. 2.**
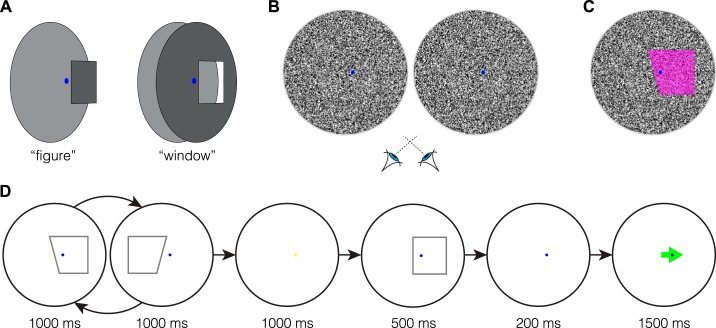
Examples of experimental stimuli and design. *A*: illustration of the 2 (figure and window) depth structures used in the experiment. *B*: stereogram of the (figure) adaptor stimulus in the binocular disparity condition designed for cross-eyed fusion. *C*: 1 frame of the adaptor stimulus in the motion parallax condition; the figure region is indicated in magenta. Pixels either inside (window) or outside (figure) the trapezoidal region were moved horizontally according to a sinusoidal function. *D*: schematic of the design; alternating presentation of adaptation figures on the left and right (adaptation sequence) followed by a test figure and an arrow pointing either left or right, each separated by blank intervals. The observers’ task was to indicate whether the orientation of the test figure flank was consistent with the direction of the proceeding arrow (anticlockwise/left or clockwise/right).

Stimuli and design were motivated by those used by von der Heydt et al. (2005). Unlike this previous study that used solid lines on a blank background, however, our stimuli consisted of pixel noise in which shapes were distinguished by either *1*) binocular disparity or *2*) motion parallax, appearing as (near) a “figure” or (far) a “window” in front of/behind a larger surface (Fig. 2*A*). Binocular disparity-defined shapes could be either near or far (±2.5 arcmin) relative to the background, with the closer plane always at 0° offset (Fig. 2*B*). Pixel (white, 198 cd/m^2^; black, 5 cd/m^2^) luminance was randomly reassigned at 10 Hz. Similarly, motion parallax-defined shapes were made to appear near or far by moving the pixels on either the inside or the outside of the trapezoidal region horizontally according to a sinusoidal function (max speed 40°/s, with an initial phase 0 or π, counterbalanced across presentations; Fig. 2*C*). To reduce nontarget cues to orientation that could be used as a reference, the stimulus region was circular (radius 4°) with a Gaussian-smoothed edge (SD 8 arcmin). Trapezoidal regions (height 3°, center width 3°) were positioned on the left or the right side of the center of the screen with the tilted edge (henceforth referred to as the flank) centered on the midline. A blue dot (radius 6 arcmin) was positioned to the left or right of center (30 arcmin) to stabilize fixation. During blank periods the stimulus region was midgray (99 cd/m^2^); the remainder of the screen was always black (5 cd/m^2^).

#### Procedure.

Runs consisted of an initial adaptation sequence (32 pairs of trapezoids, 76.8 s), followed by test trials that were separated by top-up adaptation sequences (3 pairs of trapezoids, 7.2 s; Fig. 2*D*). During the adaptation sequences, observers viewed trapezoidal figures (left and right flank tilt either [−15, 15]° or [15, −15]° in separate blocks) alternating between left- and right-side presentation (duration 1 s, interstimulus interval 0.2 s). The depth cue (binocular disparity or motion parallax) that defined the adaptor and test figures was always the same within a run, but the depth order could be either congruent (e.g., figure with figure) or incongruent (e.g., figure with window). A method of constant stimuli was used with trials consisting of a test figure (flank tilt ± [8, 4.8, 1.6]°) presented pseudorandomly, either on the left or the right of the screen center, for a duration of 0.5 s. After the test stimulus, there was a 0.2-s blank period followed by presentation of a green arrow centered on fixation, pointing left or right (1.5 s). To mitigate fixation disruption, the fixation dot always occluded the arrow. The arrow was presented at the same depth plane as the fixation dot, and its direction was selected at random.

Observers’ task was to press the “space” key when the test flank’s tilt direction (anticlockwise/left or clockwise/right) appeared to be consistent with the direction of the following arrow. This method of response was used instead of separate left/right responses to avoid systematic response bias, such as observers pressing left or right more frequently under high uncertainty. A 1-s blank interval separated adaptation and test periods to avoid a potential bias of the afterimage of the last-presented adaptation figure. For the same reason, the starting trapezoid of the adaptation sequence was alternated so that left-/right-side trapezoids were presented as the last figure on half the trials. During the blank interval preceding each trial, the fixation dot was changed from blue to yellow to prepare observers for the upcoming trial. Before adaptation runs, baseline test runs were performed in which subjects were presented with the same test stimuli without adaptation.

In *experiment 1*, we used a 2 × 2 × 2 factorial design: depth cue (motion parallax/binocular disparity) × depth (figure/window) ×depth congruence (congruent/incongruent). Adaptation and baseline runs consisted of 48 and 72 trials, respectively. For each condition, observers underwent two baseline and two adaptation runs, one for each of the left-/right-positioned fixation dots (randomized order). In each session, observers completed two conditions, for a total duration of ~60 min. To avoid carryover effects, sessions were completed on separate days and the adaptation orientation was reversed between conditions. Depth-congruent runs were completed before depth-incongruent runs, and the depth cue condition was held constant within sessions. The order of depth sign and depth cue conditions was counterbalanced across participants.

In *experiment 2*, we tested whether adaptation to one depth cue influenced objects defined by the other depth cue. We therefore ran two conditions in which the depth portrayed a (near) figure but the cues used to define the adaptor and test stimuli were incongruent. For example, an observer would adapt to motion-defined objects and was then tested with disparity-defined objects, or vice versa.

#### Task response data analysis.

For each condition, we concatenated data from the two runs and fit them with a psychometric function using the MATLAB toolbox Psignifit (Fründ et al. 2011; see http://psignifit.sourceforge.net/) to establish separate threshold, i.e., point of subjective equality, and slope values for responses corresponding to test figures presented on the left and right. To determine the bias on perceived orientation that resulted from adaptation, we calculated the difference between baseline and adaptation threshold values. To match the bias between runs with [−15, 15]° tilt adaptors across observers, we reversed the sign of bias in the [15, −15]° tilt runs. For the same reason, we reversed the sign of the bias for figures presented on the left before averaging the two, yielding a single measure of bias for each condition. Thus average bias was normalized to the −15° tilt adaptor condition.

#### Eye tracking data analysis.

A possible concern is that eye movements could influence the magnitude of the putative tilt aftereffects differently between conditions, e.g., eye movements could reduce the spatial localization of adaptation to the point of failing to detect a measurable aftereffect. Thus to test this possibility we measured observers’ eye movements during the experiment. Before analysis, eye movement data were screened to remove blinks and noisy and/or spurious recordings. To test for differences in eye position between experimental conditions, we calculated the mean and standard deviation of observers’ vertical and horizontal binocular eye position, relative to fixation, for version and vergence eye movements during adaptor and test presentation. To match eye position coordinates between runs with left- and right-side fixation, we reversed the sign of horizontal eye movements in runs in which the fixation dot was presented on the left side.

#### Data availability.

The data presented in this study are available at https://www.repository.cam.ac.uk/handle/1810/290803.

## RESULTS

### 

#### Selectivity for multiple depth cues.

Neurophysiological work has revealed orientation-tuned cells in the primary visual cortex of macaque that conditionally respond to the borders of figures. Psychophysical work with humans has demonstrated a behavioral correlate of this cellular characteristic: border ownership-dependent tilt aftereffects (von der Heydt et al. 2005). For a neural system to effectively support figure-ground segmentation in a three-dimensional environment, at least two crucial characteristics are required: *1*) sensitivity to multiple depth cues and *2*) depth order preference.

**Fig. 3.**
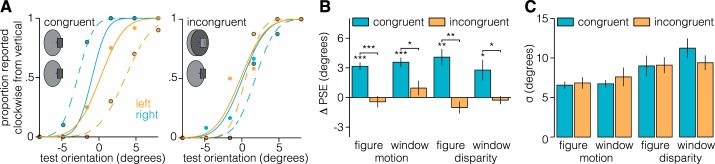
Psychophysical measurements of orientation judgments after adaptation. After adaptation to oriented stimuli defined by either motion parallax or binocular disparity, observers judged the orientation of the edge of a test stimulus that was either the same (congruent) or different (incongruent) depth order. *A*: representative example of the psychometric functions fit to 1 (naive) participant’s data in the motion-figure congruent and incongruent conditions. Solid lines and dots without outlines indicate baseline data; dashed lines and dots with black outlines indicate adaptation data. Orange and cyan dots and lines indicate data from test figures presented on the left and right sides, respectively. *B*: relative to a preadaptation baseline, observers’ point of subjective equality (PSE) was biased away from the orientation of the adaptor when the test stimulus was depth congruent (paired *t*-test; motion-near: *t*_7_ = 8.8, *P* = 4.9e^−5^, *d* = 6.2; motion-far: *t*_7_ = 7.9, *P* = 9.5e^−5^, *d* = 5.6; disparity-near: *t*_7_ = 5.3, *P* = 0.001, *d* = 3.7; disparity-far: *t*_7_ = 2.8, *P* = 0.023, *d* = 2.0), but not depth incongruent (paired *t*-test; motion-near: *t*_7_ = −0.8, *P* = 0.446, *d* = 0.6; motion-far: *t*_7_ = 1.3, *P* = 0.216, *d* = 0.9; disparity-near: *t*_7_ = −1.8, *P* = 0.120, *d* = 1.2; disparity-far: *t*_7_ = −0.7, *P* = 0.508, *d* = 0.5), with the adaptor. Furthermore, the PSE in depth-congruent conditions was significantly more biased than in corresponding depth-incongruent conditions (paired *t*-test; motion-near: *t*_7_ = 2.7, *P* = 0.029, *d* = 1.9; motion-far: *t*_7_ = 6.9, *P* = 2.3e^−4^, *d* = 4.9; disparity-near: *t*_7_ = 4.5, *P* = 0.003, *d* = 3.2; disparity-far: *t*_7_ = 2.8, *P* = 0.028, *d* = 2.0). *P < 0.05, **P < 0.01, ***P < 0.001, significant differences. *C*: the difference in bias between depth-congruent and -incongruent conditions cannot be explained by differences in the precision of judgments; we found no differences in precision (inverse of σ) between corresponding depth-incongruent/congruent conditions (paired *t*-test; motion-near: *t*_7_ = 0.7, *P* = 0.513, *d* = 0.5; motion-far: *t*_7_ = 0.8, *P* = 0.420, *d* = 0.6; disparity-near: *t*_7_ = 0.1, *P* = 0.956, *d* = 0.1; disparity-far: *t*_7_ = 1.6, *P* = 0.152, *d* = 1.3). Labels along the *x*-axis refer to the depth of the test stimulus.

Here we tested whether adaptation to figures defined by one of two primary depth cues (motion parallax or binocular disparity) produces a border ownership-dependent tilt aftereffect. We further investigated whether any adaptation effect is transferable between depth order configurations (e.g., adapt to a near object and test with a far object). We found that perceptual bias following adaptation was significantly larger when the adaptor and test stimulus depth were congruent than when they were incongruent (repeated-measures ANOVA; *F*_1,49_ = 37.6, *P* = 1.5e^−7^, *d* = 4.3; Fig. 3, *A* and *B*). Indeed, we found a significant adaptation effect for all depth-congruent conditions but failed to find an effect for any depth-incongruent condition (Fig. 3*B*). We also found no difference in the strength of the effect for different cues (*F*_1,49_ = 0.4, *P* = 0.521, *d* = 0.4) or depths (*F*_1,49_ = 4.0, *P* = 0.087, *d* = 1.4). No interactions were significant (all *P* > 0.05). These results indicate that border ownership cells are sensitive to both motion parallax and binocular disparity and that they show depth order preference. A possible concern is that we failed to detect a bias in the depth-incongruent conditions because bias estimates were less precise. Such a problem could arise because of, for example, reduced anticipation of the test stimulus due to the change in depth relative to the adaptor stimulus. However, we found no evidence for a difference in the precision of observers’ estimates between depth-congruent and depth-incongruent conditions (*F*_1,49_ = 0.04, *P* = 0.849, *d* = 0.14; Fig. 3*C*). By contrast, we found that observers’ judgments were more precise for stimuli defined by motion parallax than by binocular disparity (*F*_1,49_ = 15.6, *P* = 0.006, *d* = 2.8). No differences in the precision of estimates were observed between depth conditions (*F*_1,49_ = 4.2, *P* = 0.079, *d* = 1.4).

**Fig. 4.**
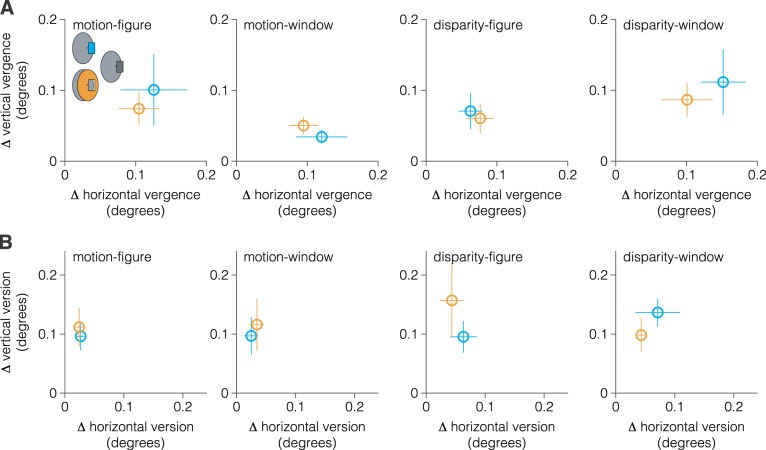
Change in binocular eye gaze position between adaptor and test stimulus presentation. To test whether our failure to detect transference of adaptation between (relatively) near and far figure borders was due to larger changes in eye gaze position between test and adaptor stimulus presentation in the incongruent condition, we compared the absolute difference in the average horizontal/vertical vergence (*A*) and version (*B*) eye movements between depth-congruent (cyan) and -incongruent (orange) conditions. We found no evidence for a larger change in binocular eye gaze position for either vergence or version eye movements. The label at the top of each plot indicates the test stimulus condition.

The border ownership-dependent tilt aftereffect is retinotopically localized to a relatively small region (~2°) (von der Heydt et al. 2005); thus another possible concern is that we failed to detect a tilt aftereffect in the incongruent conditions because observers’ gaze position moved more between adaptor and test stimulus presentation than in congruent conditions. However, we found no evidence for a larger change in binocular eye position between adaptor and test periods for either horizontal/vertical vergence (repeated-measures ANOVA; horizontal: *F*_1,49_ = 2.8, *P* = 0.159, *d* = 1.2; vertical: *F*_1,49_ = 0.7, *P* = 0.433, *d* = 0.6; Fig. 4*A*) or version (horizontal: *F*_1,49_ = 0.4, *P* = 0.541, *d* = 0.4; vertical: *F*_1,49_ = 0.3, *P* = 0.593, *d* = 0.4; Fig. 4*B*) eye movements.

#### Joint encoding of depth cues.

**Fig. 5.**
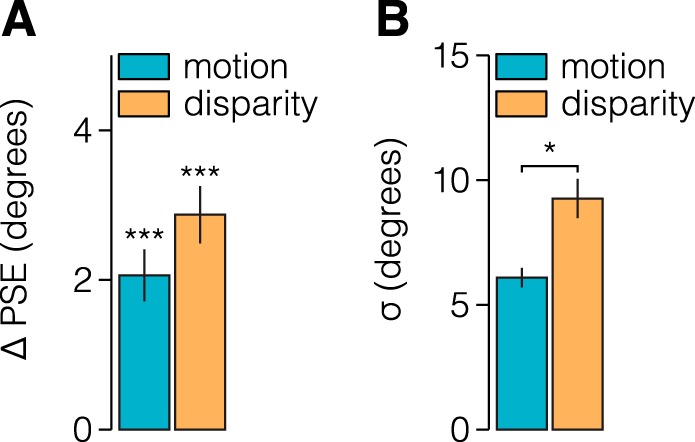
Transfer of adaptation effects between stimuli defined by motion parallax and binocular disparity. After adaptation to oriented figure defined by either motion parallax or binocular disparity, observers judged the orientation of a test figure that was defined by the other cue. *A*: relative to a preadaptation baseline, observers’ point of subjective equality (PSE) was biased away from the orientation of the adaptor for both stimuli. The shade of the bars indicates the depth cue used to define the test stimulus, whereas the opposite cue was the adaptor stimulus. *B*: consistent with the results from the previous experiment, we also found that judgments made for stimuli defined by motion were more precise than for disparity. *P < 0.05, ***P < 0.001, significant differences.

The results above suggest that the neural systems supporting border ownership are tuned to motion parallax and binocular disparity depth cues. There are two ways in which these cues may be encoded by these systems: *1*) depth cues are encoded in separate neural populations, or *2*) neurons supporting border ownership jointly encode depth cues. Consistent with the joint encoding hypothesis, previous neurophysiological work indicates that some border ownership cells combine binocular disparity and gestalt cues (Qiu and von der Heydt 2005). To determine whether the neural systems supporting border ownership encode (motion parallax and binocular disparity) depth cues separately or jointly, we tested whether the effect of adaptation to a stimulus defined by one cue was transferred to a stimulus defined by the other. In line with the hypothesis that cues are jointly encoded, we found a significant tilt aftereffect for stimuli defined by motion parallax after adaptation to stimuli defined by binocular disparity (paired *t*-test, *t*_7_ = 6.3, *P* = 3.9e^−4^, *d* = 4.4), and vice versa (*t*_7_ = 8.0, *P* = 8.8e^−5^, *d* = 5.7; Fig. 5*A*). Consistent with our previous results, we again found that judgments for stimuli defined by motion parallax were more precise (*t*_7_ = 4.1, *P* = 0.004, *d* = 2.9; Fig. 5*B*).

## DISCUSSION

Psychophysical work with human observers has shown that multiple tilt aftereffects can be induced at the same retinotopic location by adaptation to the orientation of overlapping borders of different figures (von der Heydt et al. 2005). This finding supports evidence from neurophysiological work with macaque revealing orientation-tuned border ownership cells in the primary visual cortex that conditionally respond to figure borders (Zhou et al. 2000). These findings were obtained with luminance-defined objects viewed on two-dimensional displays. However, objects in our natural environment are seldom defined in this manner; they are often signaled by multiple depth cues such as motion parallax and binocular disparity. To efficiently segment the visual environment, the neural systems supporting border ownership would be expected to have access to depth cues and to show strong depth order tuning. To test this hypothesis, here we measured border ownership-dependent tilt aftereffects for stimuli defined by two primary depth cues, motion parallax and binocular disparity. We found tilt aftereffects for both cues, which were not transferable between depth order configurations (e.g., adapting to a “figure” edge did not influence perception of a “window” edge) but were transferable between cues (e.g., adapting to motion parallax influenced perception of a subsequent object defined by binocular disparity).

The finding that a border ownership-dependent tilt aftereffect can be induced with stimuli defined by motion parallax or binocular disparity shows that the neural systems supporting border ownership have access to information from multiple depth cues. These psychophysical results provide converging evidence consistent with previous work showing that binocular disparity and gestalt cues are both encoded by border ownership cells (Qiu and von der Heydt 2005) and may indicate that border ownership cells are sensitive to a range of depth cues. The finding that tilt aftereffects were nontransferable between depth configurations shows that the neural systems supporting border ownership have strict depth order preference; an essential characteristic for establishing the depth order of objects within a scene.

Visual attention is biased toward near surfaces (West et al. 2013), suggesting that most objects that are the focus of the visual system are figurelike rather than windowlike. Thus one may expect aftereffects to be stronger for the borders of figures than windows. However, we found no evidence to support this hypothesis; there was no difference between the strength of aftereffects for figures and windows defined by either motion parallax or binocular disparity.

If the behavioral effects observed here are a result of adapting the responses of a population of border ownership cells in the human brain, it is interesting to consider how our results align with neurophysiological evidence of these cells in the macaque. For instance, Qiu and von der Heydt (2005) found that only just over half of border ownership cells tuned to binocular disparity were also depth order selective. This seems (at least partially) at odds with the strict depth order preference of the tilt aftereffects. That is, if a considerable proportion (40%) of border ownership cells are not selective for depth order, it may have been reasonable to have expected to find transference of adaptation between depth order conditions; yet we found no evidence for this. If the behavioral effects observed here were produced by adapting border ownership cells, this may suggest that depth order selectivity is more prevalent in these cells than previously estimated. However, the proportion of depth order-selective border ownership cells in the macaque primary visual cortex may not be representative of those in the human, and the relationship between the proportion of these cells and their behavioral consequences may not be linear. Furthermore, in the present experiments observers likely attended to the figures, whereas in the macaque experiments the subjects’ attention was likely focused on the fixation task (Qiu and von der Heydt 2005); thus differences across species may be due to differences in attentional allocation across tasks (Fang et al. 2009; Qiu et al. 2007). This cross-species incongruence underscores the importance of psychophysical evidence in establishing the perceptual influence of stimuli as processed by an entire neural system.

The finding that adaptation effects could be transferred between stimuli defined by either motion parallax or binocular disparity indicates that the neural systems supporting border ownership jointly encode these depth cues. Border ownership mechanisms serve to bind contour features to larger entities and have a high degree of orientation and spatial position specificity. In contrast, the higher-level figure-ground mechanisms first demonstrated by Lamme (1995) that support the response modulation observed between figure and ground regions are, among other differences summarized by von der Heydt (2015), less spatially precise and occur independently of the tuning properties of neurons (Zipser et al. 1996). The present findings suggest that the responses of a subpopulation of neurons tuned to a particular orientation were reduced contingent on specific left/right and near/far positional characteristics of the figure. This specificity is characteristic of border ownership mechanisms, indicating that integration of motion parallax and binocular disparity signals is supported by early border ownership neural systems rather than later figure-ground processes. The present findings cannot distinguish whether integration is performed by border ownership cells or by higher-level “groupings cells” (Craft et al. 2007). However, the finding that binocular disparity and gestalt cues are integrated by border ownership cells (Qiu and von der Heydt 2005) implicates this earlier stage as a likely candidate.

Functional MRI (fMRI) work with humans indicates that motion parallax and depth cues are combined in area V3B/KO (Ban et al. 2012). By contrast, neurophysiological work has identified border ownership cells in area V2 of the macaque cortex that jointly encode binocular disparity and gestalt cues (Qiu and von der Heydt 2005). Our results may therefore indicate that motion parallax and binocular disparity signals are combined before V3B/KO. For instance, the neural systems that combine the signals evoked by these cues may be present as early as V2 and culminate in higher cortical areas such as V3B/KO, where they are sufficiently prevalent to identify with fMRI. Indeed, fMRI work has shown that the conjunction of binocular disparity and motion direction cues can be decoded from BOLD activity by multivoxel pattern analysis as early as V2 (Seymour and Clifford 2012), providing further support that these cues may be combined as early as V2. Alternatively, these results may indicate that border ownership cells, which combine motion parallax and binocular disparity, are present in V3B/KO. Given that neurophysiological work has exclusively found evidence for border ownership cells in ventral regions (V2 and V4), the presence of these cells in dorsal area V3B/KO would suggest that they play a more ubiquitous role in visual processing than previously thought. 

Several models have been proposed to capture the mechanism by which border ownership systems perform figure-ground segmentation, which can be broadly categorized as feedforward (Sakai and Nishimura 2006; Supèr et al. 2010), lateral (Zhaoping 2005), or feedback (Craft et al. 2007; Jehee et al. 2007) models; see Williford and von der Heydt (2013) for a review. Our data provide new challenges for these models, as they will need to incorporate the capacity of border ownership systems to jointly encode multiple depth cues with a high prevalence of depth order selectivity. Similarly, multiple models exist for describing the way in which the brain combines cues (Ohshiro et al. 2011; Rideaux and Welchman 2018); given that border ownership mechanisms appear to combine depth cues, future work may also investigate how border ownership cells combine cues and whether it can be described by an existing model, e.g., maximum-likelihood estimation (Bülthoff and Mallot 1988) or Bayesian (Knill and Pouget 2004) frameworks.

Previous work demonstrated a behavioral correlate of border ownership with two-dimensional luminance-defined objects (von der Heydt et al. 2005). The present study exploits this correlate to show that the neural systems supporting border ownership in humans jointly encode multiple depth cues with strong depth order selectivity. These results provide compelling evidence for the capacity of border ownership mechanisms to support figure-ground segmentation in natural visual environments. Furthermore, these data provide an important future question for neurophysiological work to pursue, that is, the nature and locus of border ownership cells that combine motion parallax and binocular disparity signals.

## GRANTS

This work was supported by the Leverhulme Trust (ECF-2017-573 to R. Rideaux), the Isaac Newton Trust [17.08(o) to R. Rideaux], and the Wellcome Trust (095183/Z/10/Z to R. Rideaux as a postdoctoral fellow of Andrew E. Welchman) and by the National Health and Medical Research Council of Australia (CJ Martin Fellowship APP1091257 to W. J. Harrison).

## DISCLOSURES

No conflicts of interest, financial or otherwise, are declared by the authors.

## AUTHOR CONTRIBUTIONS

R.R. and W.J.H. conceived and designed research; R.R. performed experiments; R.R. analyzed data; R.R. interpreted results of experiments; R.R. prepared figures; R.R. drafted manuscript; R.R. and W.J.H. edited and revised manuscript; R.R. and W.J.H. approved final version of manuscript.

## ENDNOTE

At the request of the authors, readers are herein alerted to the fact that additional materials related to this manuscript may be found at the institutional Web site of the authors, which at the time of publication they indicate is: https://www.repository.cam.ac.uk/handle/1810/290803. These materials are not a part of this manuscript and have not undergone peer review by the American Physiological Society (APS). APS and the journal editors take no responsibility for these materials, for the Web site address, or for any links to or from it.
